# Influence of Human Blood Contamination on Microhardness of Glass-Ionomer Cements and Glass-Hybrid Material

**DOI:** 10.3390/ma18174075

**Published:** 2025-08-30

**Authors:** Katarina Franić, Ana Brundić, Jurica Matijević, Ana Ivanišević, Ivana Miletić, Anja Baraba

**Affiliations:** 1 University of Zagreb, School of Dental Medicine, Gundulićeva 5, 10000 Zagreb, Croatia; kfranic@sfzg.unizg.hr (K.F.); abrundic@sfzg.unizg.hr (A.B.); 2 University of Zagreb, School of Dental Medicine, Department of Endodontics and Restorative Dentistry, Gundulićeva 5, 10000 Zagreb, Croatia; matijevic@sfzg.unizg.hr (J.M.); miletic@sfzg.unizg.hr (I.M.)

**Keywords:** bioactive restorative materials, mechanical properties, thermocycling, Vickers hardness test

## Abstract

The aim of this study was to evaluate the effect of human blood contamination, before and after hardening of the materials, on microhardness of high-viscosity Fuji IX GP Extra (Fuji IX) and resin-modified Fuji II LC (Fuji II) glass-ionomer cement (GIC) and glass-hybrid material EQUIA Forte HT (EQUIA), with and without protective coating EQUIA Forte Coat (Coat), before and after thermocycling. Four groups (n = 40): 1. Fuji IX; 2. Fuji II; 3. EQUIA and 4. EQUIA + Coat were further subdivided into 3 subgroups: (1) Control; (2) blood contamination before hardening; (3) blood contamination after hardening, resulting in a total of 12 groups of 10 samples each. Samples were prepared using teflon molds (5 mm × 2 mm). Microhardness was measured using a Vickers microhardness tester before and after thermocycling (10,000 cycles), and data were statistically analyzed (Kolmogorov–Smirnov test, ANOVA, Scheffe’s test). In the control groups, the highest microhardness was measured for EQUIA+Coat before thermocycling (70.71 ± 8.79) and after thermocycling (68.6 ± 7.65). Within the groups exposed to blood after hardening, the highest microhardness was recorded in the thermocycled EQUIA+Coat group (73.07 ± 8.85). Blood contamination before hardening negatively affected the microhardness of Fuji II, Fuji IX, and EQUIA+Coat. Exposure to blood after hardening increased the microhardness of Fuji IX and EQUIA, thermocycled Fuji IX and thermocycled EQUIA + Coat samples.

## 1. Introduction

The need for biocompatible and bioactive dental materials has driven significant scientific advancements, particularly in the second half of the 20th century. With these goals in mind, Wilson and Kent developed glass ionomer cement (GIC) in 1972, and by 1973, McLean had recommended its clinical application in dental practice [1]. Although GICs offer several advantages over composite resins, such as chemical bonding to tooth structure, thermal expansion similar to dental tissues, fluoride release, and biocompatibility, no material fully replicates the properties of enamel and dentin.

Due to limitations such as brittleness, fracture susceptibility, and generally weaker mechanical properties, continuous improvements have been made to the composition and performance of GICs since their invention. High-viscosity GICs were introduced in the mid-1990s [2] to enhance the mechanical properties of conventional formulations [3]. The main distinction lies in their increased powder-to-liquid ratio and reduced particle size [4], which improves saturation of the liquid component and results in greater density, enhanced abrasion resistance, and improved mechanical performance [5].

Resin-modified glass ionomer cements (RMGIC) became available in 1991, aiming to combine the advantages of both composites and conventional GICs. In addition to powder and acid, they contain a resinous monomer component and an appropriate polymerization initiator. The most common monomer used is 2-hydroxyethyl methacrylate (HEMA), while camphorquinone typically serves as the initiator. In some materials, the polyalkenoic acid component is modified with unsaturated vinyl side chains that participate in polymerization [6].

Despite these enhancements, GICs were still not widely considered suitable for long-term restorations in posterior teeth [7]. In 2007, GC Corporation (Tokyo, Japan) introduced the EQUIA restorative system, combining high-viscosity GIC material with a nano-filled resin coating (EQUIA Coat). The nanoparticles in the coating improve mechanical performance, making the material suitable for long-term posterior restorations. The coating also enhances the esthetic properties of the micro-laminated GIC.

In 2014, GC launched the EQUIA Forte system, followed by EQUIA Forte HT in 2019. These systems, classified as glass hybrid materials, feature a modified glass matrix containing filler particles of varying sizes, including highly reactive, smaller glass particles. Additionally, they incorporate high-molecular-weight polyacrylic acid, which stabilizes the material’s core and improves its mechanical properties, making it more appropriate for use in posterior restorations [8,9]. The EQUIA Forte HT system consists of EQUIA Forte HT Fil and EQUIA Forte Coat. The coat contains evenly distributed 40 nm silica filler particles, which improve wear resistance. It also includes a highly reactive monomer that, according to the manufacturer, increases the material’s hardness by approximately 35%. The coating forms a 35–40 µm thick layer, sealing surface porosities and producing a smooth restoration [10].

The mechanical properties of a restorative material depend on both its composition and the conditions under which it is applied. Microhardness testing is commonly used to evaluate mechanical performance. Depending on the scale and applied load, hardness tests are categorized as macro-, micro-, or nano-hardness tests. Although microhardness and nanohardness do not reflect different material properties, they differ in the scale of the indentation measurement. Microhardness reflects a material’s resistance to indentation and correlates with resistance to occlusal forces. In dental research, the most widely used methods for measuring microhardness are the Vickers and Knoop techniques. These methods calculate microhardness based on the size of the indentation and the applied force [11,12]. In the Knoop method, indentations can reach lengths of up to 20 µm, while the Vickers method is less sensitive to plastic deformation, offering greater precision for elastic materials like GICs and glass-hybrids [13]. Ideally, a restorative material should demonstrate high microhardness, as this correlates with better resistance to wear in the oral environment, where it is continually exposed to saliva, food, beverages, and occlusal contacts [12,14].

The presence of blood generally has a detrimental effect on the properties of dental materials [15]. In composite resins, commonly used for direct restorations, blood contamination can reduce bonding strength by creating a thin layer on the tooth surface, preventing proper penetration of adhesives into dentinal tubules and hindering the formation of a strong bond [16]. Similarly, blood and saliva contamination have been shown to reduce micro-shear bond strength between RMGIC and resin composite [17].

According to the available literature, the influence of human blood contamination on the mechanical properties of different GIC and glass-hybrid materials remains insufficiently studied.

The objectives of this study were to: (1) Compare the baseline microhardness of a high-viscosity glass ionomer cement (Fuji IX GP Extra), a resin-modified glass ionomer cement (Fuji II LC), and a glass-hybrid material (EQUIA Forte HT) with and without a protective coating (EQUIA Forte Coat); (2) Evaluate the effect of blood contamination before hardening on the microhardness of these materials; (3) Evaluate the effect of blood contamination after hardening on the microhardness of these materials; (4) Determine the influence of thermocycling (aging) on microhardness across all experimental conditions.

## 2. Materials and Methods

### 2.1. Preparation of Samples

**Figure 1:**
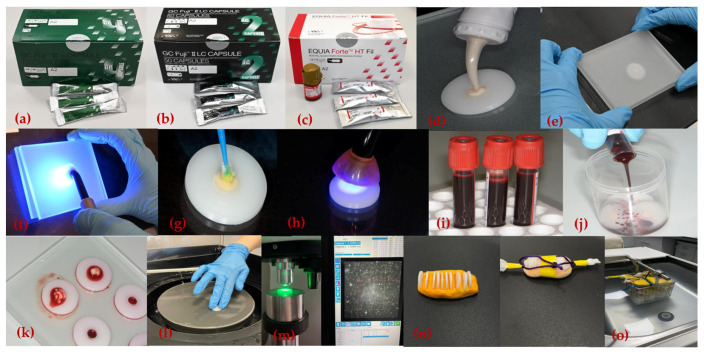
(**a**) Fuji IX GP Extra; (**b**) Fuji II LC; (**c**) EQUIA Forte HT and EQUIA Forte Coat; (**d**) placing mixed material into teflon mold; (**e**) teflon mold with mixed material covered with a glass plate, applying moderate pressure to ensure proper condensation and a flat surface; (**f**) light curing Fuji II LC; (**g**) coating the surface of EQUIA Forte HT; (**h**) light curing EQUIA Forte Coat; (**i**) human blood in vials; (**j**) exposing samples to human blood after hardening; (**k**) exposing samples to human blood before hardening; (**l**) polishing of samples; (**m**) microhardness testing; (**n**) smples prepared for thermocycling; (**o**) thermocycling.

The study was approved by the Ethics Committee of the School of Dental Medicine in Zagreb (05-PA-2-25/2024). Three encapsulated materials were used: (1) Fuji IX GP Extra A2; (2) Fuji II LC, A2; (3) EQUIA Forte HT, A2, with or without coating (EQUIA Forte Coat), (Table 1, Figure 1a–c). A total of 12 groups (4 control and 8 experimental groups) were prepared, each consisting of 10 samples (n = 10).

A total of four control groups (1. Fuji IX GP Extra, 2. Fuji II LC, 3. EQUIA Forte HT, 4. EQUIA Forte HT+EQUIA Forte Coat) were mixed and prepared according to the manufacturer’s instructions. Custom-made teflon molds (5 mm in diameter and 2 mm in height) were used to prepare the samples. The molds were placed on a glass base, filled with a slight excess of material (Figure 1d), and covered with a glass plate, applying moderate pressure to ensure proper condensation and a flat surface (Figure 1e). In the Fuji II LC group, the material was light-cured for 20 s on each side using high power photopolymerization at an output of 1400 mW/cm^2^ (D-Light Pro, GC Corporation, Tokyo, Japan) (Figure 1f). In the EQUIA Forte HT + Coat group, after the material had set, the surface of each sample was coated on both sides with EQUIA Forte Coat (Figure 1g) and light-cured for 20 s per side using the same polymerization lamp (Figure 1h). All samples were stored in plastic containers filled with phosphate-buffered saline (PBS).

### 2.2. Exposure of Samples in Experimental Groups to Human Blood

Human blood for the study was obtained through voluntary donation by one of the researchers and collected by a qualified medical professional (Figure 1i). The next four groups were prepared identically to the control groups, but after setting for 5 min, samples were immersed in 20 mL of freshly drawn human blood for 10 min (Figure 1j). The samples of the experimental groups described (5. Fuji IX GP Extra + blood after hardening, 6. Fuji II LC + blood after hardening, 7. EQUIA Forte HT + blood after hardening, 8. EQUIA Forte HT + EQUIA Forte Coat + blood after hardening) were then rinsed with distilled water and stored in PBS. The final four experimental groups (9. Fuji IX GP Extra + blood before hardening, 10. Fuji II LC + blood before hardening, 11. EQUIA Forte HT + blood before hardening, and 12. EQUIA Forte HT+EQUIA Forte Coat + blood before hardening) were exposed to human blood before material hardening. Blood (0.1 mL) was first applied to the glass slab, onto which the molds were placed. The mixed materials were inserted into the molds, and blood was added both beneath (0.1 mL) and above the material surface (0.1 mL) (Figure 1k). A glass plate was placed over the filled molds with moderate pressure to ensure compaction and surface uniformity. Fuji II LC samples were then light-cured as previously described. For the EQUIA Forte HT + Coat group, after setting, the coating was applied to both surfaces and light-cured for 20 s per side. After 10 min, all samples were rinsed with distilled water and stored in PBS. All samples were incubated in PBS at 37 °C for 7 days (ES 120, Nuve, Ankara, Turkey) and subsequently polished using abrasive papers, progressing from coarse to fine grit, with a mechanical polishing device (Minitech 250 DP1, Presi, Eybens, France), (Figure 1l).

### 2.3. Vickers Microhardness Test and Thermocycling

Microhardness was measured using a Vickers microhardness tester (KB Prüftechnik GmbH, Hochdorf-Assenheim, Germany), applying a 1 kg load for 10 s. Five indentations were made on each sample, and values were recorded in Vickers hardness units (HV), (Figure 1m). After initial measurements, samples were thermocycled to simulate aging. Each sample was embedded in addition silicone (Elite HD + Putty Soft, Zhermack, Italy), wrapped in medical gauze, and labeled with a color-coded ribbon for identification (Figure 1n). Thermocycling was performed using thermocycler (1100/1200, SD Mechatronik, Feldkirchen-Westerham, Germany), alternating between 5 °C and 55 °C water baths for 30 s each over 10,000 cycles (Figure 1o). Microhardness measurements were then repeated.

### 2.4. Statistical Analysis

For a medium effect size (Cohen’s *f* = 0.25) in a three-way ANOVA, including the possibility of testing interactions, a sample size of *n* = 10 per group provides a statistical power of approximately 0.80–0.85, which is considered both statistically and methodologically acceptable. Each sample underwent five Vickers hardness measurements, which were averaged to obtain a single representative value. This approach increased the reliability and precision of the measurement without inflating the sample size or violating the assumption of independence.

Statistical analysis was performed using parametric methods. Normality was confirmed with the Kolmogorov–Smirnov test. Group results were presented as means and standard deviations. Differences between groups were analyzed using three-way ANOVA, followed by Scheffé’s post hoc test for pairwise comparisons. A *p*-value of less than 0.05 was considered statistically significant.

## 3. Results

### 3.1. Microhardness of Control Groups Before Thermocycling

Among the control groups, the highest microhardness before thermocycling was measured for EQUIA Forte HT+EQUIA Forte Coat (70.71 ± 8.79), which was statistically significantly higher compared to the other materials (*p* = 0.003 for Fuji IX GP Extra, *p* < 0.001 for Fuji II LC and EQUIA Forte HT), (Table 2). Fuji IX GP Extra demonstrated slightly lower microhardness (64.23 ± 9.66), which was statistically significantly higher compared to the two materials with the lowest microhardness, EQUIA Forte HT (48.60 ± 9.45) and Fuji II LC (45.63 ± 5.22), between which no statistically significant difference was found (*p* = 0.383), (Table 2).

### 3.2. Microhardness of Control Groups After Thermocycling

After thermocycling, EQUIA Forte HT+EQUIA Forte Coat again exhibited the highest microhardness among the control groups (68.57 ± 7.65), with a statistically significant difference (*p* < 0.001), (Table 2). No significant difference in microhardness was observed between EQUIA Forte HT and Fuji IX GP Extra (*p* = 0.911), while Fuji II LC showed the lowest microhardness (49.66 ± 8.01), which was significantly lower than all other materials (*p* < 0.001) (Table 2).

Overall, thermocycling had a statistically significant effect on the microhardness of the tested materials (*p* < 0.001). Specifically, EQUIA Forte HT and Fuji II LC demonstrated significantly increased microhardness after thermocycling (*p* < 0.001), whereas no statistically significant change was observed in EQUIA Forte HT + EQUIA Forte Coat or Fuji IX GP Extra (*p* > 0.05) (Table 2).

### 3.3. Microhardness of Experimental Groups Before Thermocycling

**Figure 2:**
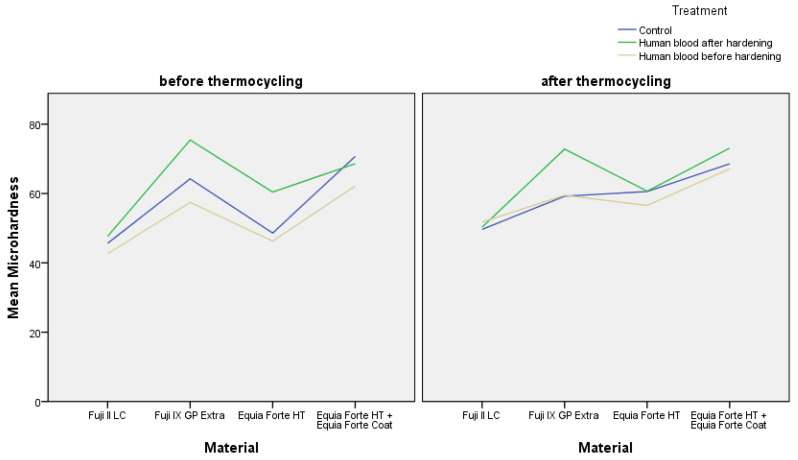
Mean microhardness values of tested materials before and after thermocycling based on treatment: control, exposure to human blood before and after hardening.

Among the experimental groups in which materials were exposed to human blood either before or after hardening, but prior to thermocycling, for Fuji II LC, the differences in measured microhardness between samples not exposed to human blood and those exposed to human blood after hardening were not statistically significant (*p* > 0.05). However, samples exposed to human blood before hardening exhibited statistically significantly lower microhardness (42.65 ± 4.21), (*p* = 0.01), (Table 2, Figure 2). For Fuji IX GP Extra, differences before thermocycling were statistically significant, as the microhardness of samples exposed to human blood before hardening was significantly lower (57.45 ± 8.39), (*p* < 0.001 for all comparisons), while the microhardness of samples exposed to human blood after hardening was significantly higher (75.43 ± 5.57) compared to the control group (*p* < 0.01), (Table 2, Figure 2). For EQUIA Forte HT, the highest microhardness was measured in the group exposed to blood after hardening (60.42 ± 7.40), (*p* < 0.001), while there was no statistically significant difference in microhardness between the control group and the group exposed to human blood before hardening (*p* > 0.05), (Table 2, Figure 2). For EQUIA Forte HT+EQUIA Forte Coat, exposure to human blood before hardening negatively affected microhardness, as a statistically significantly lower microhardness was measured (62.15 ± 7.79), (*p* < 0.001) compared to the control group and the group exposed to human blood after hardening, between which there was no statistically significant difference (*p* > 0.05), (Table 2, Figure 2). When comparing materials exposed to human blood before hardening and before thermocycling, no statistically significant differences were found between Fuji II LC (42.65 ± 4.21) and EQUIA Forte HT (46.26 ± 7.51) (*p* > 0.05), while differences in microhardness for all other materials were statistically significant (*p* < 0.001) (Table 2, Figure 2). When comparing materials exposed to human blood after hardening and before thermocycling, the differences in microhardness were statistically significant for all tested materials (*p* < 0.001) (Table 2, Figure 2).

### 3.4. Microhardness of Experimental Groups After Thermocycling

When comparing the relationship between materials and blood contamination before and after thermocycling, it was evident that thermocycling increased the differences in microhardness between Fuji IX GP Extra and EQUIA Forte HT+EQUIA Forte Coat in groups exposed to blood after hardening. For EQUIA Forte HT, the group exposed to blood after hardening did not differ significantly from the other EQUIA Forte HT groups after thermocycling. Additionally, thermocycling reduced the differences observed among Fuji II groups. Prior to thermocycling (Figure 2), the differences between treatments for Fuji II LC were statistically significant, whereas after thermocycling, no statistically significant differences were observed between the groups (*p* > 0.05) (Table 2, Figure 2). For Fuji IX GP Extra, after thermocycling, there was no statistically significant difference between the control group and the group exposed to blood before hardening (*p* = 0.984) (Table 2, Figure 2). Similarly, for EQUIA Forte HT, after thermocycling, there were no statistically significant differences between groups (*p* > 0.05) (Table 2, Figure 2). In contrast, for EQUIA Forte HT+EQUIA Forte Coat after thermocycling, the difference in microhardness was statistically significant between the group exposed to blood after hardening and the other groups (*p* = 0.001 for the group exposed to blood before hardening and *p* = 0.013 for the control group).

## 4. Discussion

This in vitro study demonstrates that the timing of blood contamination has different effects on the microhardness of glass-ionomer and glass-hybrid restorative materials. Blood exposure before hardening significantly reduced the microhardness of Fuji II LC, Fuji IX GP Extra, and Equia Forte HT with EQUIA Forte Coat. In contrast, blood exposure after hardening significantly increased the microhardness of Fuji IX GP Extra, EQUIA Forte HT, thermocycled Fuji IX, and thermocycled EQUIA Forte HT with EQUIA Coat.

The longevity and clinical success of modern restorative materials depend largely on their ability to withstand conditions in the oral environment [18]. Within the oral cavity, restorative materials are exposed to masticatory and parafunctional forces, dietary influences, moisture, and frequent temperature fluctuations. These factors collectively impact the long-term durability and performance of restorations [19]. Although clinical studies remain the gold standard for evaluating the effectiveness and longevity of restorative materials in dentistry [20, 21], conducting controlled in vivo research is both time-consuming and costly. Furthermore, if clinical trials are not properly designed in accordance with current guidelines for the planning, implementation, and assessment of direct and indirect restorations, the resulting data may lack validity and be excluded from meta-analyses, critical tools for synthesizing clinical evidence. In contrast, in vitro studies allow for faster data collection on larger sample sizes and enable partial simulation of clinical conditions, offering preliminary insights into the expected in vivo behavior of tested materials. All restorative materials are subject to a range of laboratory tests, among which the evaluation of mechanical properties and interaction with hard dental tissues is particularly important. One such key mechanical property is microhardness, which provides insight into a material’s resistance to surface wear, especially in occlusal regions exposed to heavy functional loading [22]. In this study, the in vitro microhardness of a high-viscosity glass ionomer cement (Fuji IX GP Extra), a resin-modified glass ionomer cement (Fuji II LC), and a glass-hybrid material (EQUIA Forte HT) with and without a protective coating (EQUIA Forte Coat) was assessed following exposure to human blood either immediately after mixing, before hardening, or after the setting of the material. The null hypothesis, stating that exposure to human blood would not result in significant differences in the microhardness of the tested materials, was rejected, as the results demonstrated statistically significant differences in microhardness depending on whether blood contamination occurred before or after material hardening.

Among the control groups that were not exposed to human blood, the highest microhardness was recorded for the coated glass-hybrid material (EQUIA Forte HT+EQUIA Forte Coat). This result was somewhat expected, given that glass-hybrid materials contain highly reactive glass particles uniformly distributed within the powder phase. Additionally, the polyacrylic acid used in these materials has an optimized molecular weight to enhance the availability of carboxyl groups, promoting a more efficient acid-base reaction, greater polysalt formation, and more extensive crosslinking within the cement matrix. Moshaverinia et al. [23] also demonstrated that glass-hybrid materials exhibit higher microhardness values compared to high-viscosity GIC (Fuji IX GP), consistent with the findings of this study. The lowest microhardness values were recorded in the uncoated glass-hybrid material (EQUIA Forte HT) and the resin-modified GIC (Fuji II LC). Bala et al. [24] also reported that resin-modified GICs exhibit lower microhardness compared to other GICs. The lower microhardness of the uncoated glass hybrid material is not unexpected, as the 35–40 μm-thick protective coating layer is designed to infiltrate surface porosities and irregularities, shielding the material from moisture and enhancing wear resistance during the early stages of setting. Alqasabi et al. [25] and Handoko et al. [26] also confirmed that nanoparticle-filled protective coatings, such as the one used in this study, significantly improve the microhardness of glass-hybrid materials, supporting our findings. Interestingly, Faraji et al. [27] and Ryu et al. [28] reported contrasting results, suggesting that protective coatings may reduce surface hardness compared to uncoated GICs and glass hybrid materials. These discrepancies may be attributed to differences in sample preparation. In contrast to Faraji et al. and Ryu et al., the present study, as well as those by Alqasabi et al. and Handoko et al., involved polishing the samples prior to microhardness testing, which likely ensured that the hardness measurements reflected the intrinsic properties of the material rather than the softer, unpolished coating layer.

Contamination of restorative materials with blood can occur due to gingival bleeding, trauma to the oral soft tissue, or pulpal hemorrhage [29,30]. The high protein content of blood allows it to form a film on the surface of hard dental tissues, while macromolecules like fibrinogen and platelets can hinder material adhesion, thereby negatively affecting the bond strength to enamel and dentin [31]. Blood contamination may also interfere with the setting reaction of restorative materials, subsequently impacting their mechanical properties. Specifically, proteins present in blood may disrupt crystal nucleation and promote porosity, leading to compromised material properties when exposure occurs during the setting phase [32,33].

In this study, exposure of Fuji II LC to blood before hardening resulted in a significant reduction in microhardness, while samples exposed to blood after setting showed no significant differences compared to the control group prior to thermocycling. Generally, exposure of GICs to blood has been associated with ion release and loss, potentially compromising mechanical integrity [34]. Resin-modified GICs, which harden through both light-induced polymerization and acid-base reactions, benefit from the polymerization of HEMA in their composition. This polymerization helps stabilize the material by regulating water balance and promoting polyacrylate salt formation [35,36]. However, if blood contamination occurs before hardening and before light polymerization, the unpolymerized monomers are vulnerable to disruption by water, which makes up approximately 90% of blood plasma, thus impairing the setting reaction and weakening the mechanical properties. For Fuji IX GP Extra, the highest microhardness was observed in samples exposed to blood after hardening, while the lowest values were recorded in those contaminated during setting. A similar trend was observed for EQUIA Forte HT, with statistically significant differences between the exposure conditions. Shimada et al. [37] described a phenomenon known as secondary maturation, wherein uncoated GICs and glass-hybrid materials absorb calcium ions from saliva, contributing to enhanced surface microhardness. In the present study, samples were stored in phosphate-buffered saline (PBS), which lacks calcium ions. However, blood contains approximately 1% calcium, which may have facilitated this secondary maturation process and contributed to the observed increase in microhardness.

Artificial aging through thermocycling simulates the thermal stresses that restorative materials are subjected to due to temperature fluctuations in the oral environment. Although the frequency of these temperature cycles in vivo remains undetermined, it is proposed that such cycles might occur between 20 and 50 times in a day [38]. Therefore, approximately 10,000 thermal cycles are considered equivalent to one year of clinical service [38], and for this reason, the same number of cycles was chosen in the present study. In this study, thermocycling revealed that the highest post-aging microhardness was observed for EQUIA Forte HT + EQUIA Forte Coat, whereas Fuji II LC demonstrated the lowest values. After thermocycling, no statistically significant differences in microhardness were observed between Fuji IX GP Extra and EQUIA Forte HT, which is consistent with the known time-dependent improvement in mechanical properties observed in materials that set via acid-base reactions [39]. These findings suggest that exposure to blood after setting, particularly in glass-hybrid materials, may enhance calcium ion uptake, supporting secondary maturation and improved microhardness over time. This highlights the importance of proper clinical handling and the potential benefit of surface coatings in enhancing the durability and long-term performance of restorative materials.

While this in vitro study provides valuable insights, several limitations should be considered when interpreting the findings. In this study, conditions of blood exposure, storage in phosphate-buffered saline, and thermocycling were standardized, which allowed valid comparisons; however, the oral environment is far more complex, with variable pH, saliva composition, and mechanical loading. Although the observed positive effect of blood contamination after hardening on microhardness is noteworthy, its impact on the long-term clinical performance of the tested materials remains uncertain. Future studies should evaluate the influence of blood contamination on a broader range of clinically relevant mechanical properties, including flexural strength, wear resistance, and fracture toughness. Furthermore, incorporating mechanical cyclic loading and longer-term storage conditions that more closely resemble the oral environment would help confirm the clinical relevance of the present findings.

## 5. Conclusions

When the materials were not exposed to human blood, the highest microhardness values, both before and after thermocycling, were observed in the glass-hybrid material with a protective coating (EQUIA Forte HT + EQUIA Forte Coat). Blood contamination before hardening negatively affected the microhardness of the resin-modified GIC (Fuji II LC), the high-viscosity GIC (Fuji IX GP Extra), and the glass-hybrid material with a protective coating (EQUIA Forte HT + EQUIA Forte Coat). In contrast, this contamination had no effect on the microhardness of the glass-hybrid material without the protective coating (EQUIA Forte HT). Blood contamination after hardening led to an increase in microhardness for high-viscosity GIC (Fuji IX GP Extra), both before and after thermocycling, glass-hybrid material without a protective coating (EQUIA Forte HT) before thermocycling, and glass-hybrid material with a protective coating (EQUIA Forte HT + EQUIA Forte Coat) after thermocycling.

## Figures and Tables

**Table 1:** Type, manufacturer, and composition of materials used in the study.

Material	Type of Material	Manufacturer	Composition
Fuji II LC	Resin-modified GIC	GC (Tokyo, Japan)	Liquid: 24% polyacrylic acid; 25% distilled water; 35% HEMA; 6% tartaric acid; 0.1% camphorquinone; 2, 2, 4 TMHEDC; TEGDMA. * Powder: fluoroaluminosilicate glass
Fuji IX GP Extra	High-viscosity GIC	GC (Tokyo, Japan)	Liquid: 50% distilled water; 40% polyacrylic and tartaric acid; 10% polybasic carboxylic acid. Powder: 95% fluorosilicate glass; 5% polyacrylic acid
EQUIA Forte HT	Glass-hybrid material	GC (Tokyo, Japan)	Liquid: 40-aqueous solution of polyacrylic acid. Powder: 95% fluorosilicate glass; 5% polyacrylic acid
EQUIA Forte Coat	Low viscosity nanofilled resin	GC (Tokyo, Japan)	40–50% Methyl methacrylate 10–15% colloidal silica 0.09% camphorquinone 20–40% urethane methacrylate 1–5% phosphate ester monomer

* 2, 2, 4 TMHEDC: 2, 2, 4 trimethyl hexamethylene dicarbonate, TEGDMA: triethylene glycol dimethacrylate.

**Table 2:** Mean values and standard deviations of microhardness for all groups before and after thermocycling (50 measurements per group, n = 10 per group).

Material	Treatment	Thermocycling	Mean Value	Standard Deviation
Fuji II LC	control group	before	45.63	5.22
		after	49.66	8.01
	blood after hardening	before	47.65	4.97
		after	50.28	7.49
	blood before hardening	before	42.65	4.21
		after	51.76	6.36
Fuji IX GP Extra	control group	before	64.23	9.66
		after	59.23	7.45
	blood after hardening	before	75.43	5.57
		after	72.81	7.36
	blood before hardening	before	57.45	8.39
		after	59.49	7.36
EQUIA Forte HT	control group	before	48.60	9.45
		after	60.57	12.63
	blood after hardening	before	60.42	7.40
		after	60.69	7.55
	blood before hardening	before	46.26	7.51
		after	56.56	7.16
EQUIA Forte HT+ EQUIA Forte Coat	control group	before	70.71	8.79
		after	68.57	7.65
	blood after hardening	before	68.60	6.59
		after	73.07	7.85
	blood before hardening	before	62.15	7.79
		after	67.10	7.15

## Data Availability

The original contributions presented in this study are included in the article material. Further inquiries can be directed to the corresponding authors.
